# Genomic prediction with parallel computing for slaughter traits in Chinese Simmental beef cattle using high-density genotypes

**DOI:** 10.1371/journal.pone.0179885

**Published:** 2017-07-19

**Authors:** Peng Guo, Bo Zhu, Lingyang Xu, Hong Niu, Zezhao Wang, Long Guan, Yonghu Liang, Hemin Ni, Yong Guo, Yan Chen, Lupei Zhang, Xue Gao, Huijiang Gao, Junya Li

**Affiliations:** 1 Laboratory of Molecular Biology and Bovine Breeding, Institute of Animal Science, Chinese Academy of Agricultural Sciences, Beijing, China; 2 College of Computer and Information Engineering, Tianjin Agricultural University, Tianjin, China; 3 Animal Science and Technology College, Beijing University of Agriculture, Beijing, China; China Agricultural University, CHINA

## Abstract

Genomic selection has been widely used for complex quantitative trait in farm animals. Estimations of breeding values for slaughter traits are most important to beef cattle industry, and it is worthwhile to investigate prediction accuracies of genomic selection for these traits. In this study, we assessed genomic predictive abilities for average daily gain weight (ADG), live weight (LW), carcass weight (CW), dressing percentage (DP), lean meat percentage (LMP) and retail meat weight (RMW) using Illumina Bovine 770K SNP Beadchip in Chinese Simmental cattle. To evaluate the abilities of prediction, marker effects were estimated using genomic BLUP (GBLUP) and three parallel Bayesian models, including multiple chains parallel BayesA, BayesB and BayesCπ (PBayesA, PBayesB and PBayesCπ). Training set and validation set were divided by random allocation, and the predictive accuracies were evaluated using 5-fold cross validations. We found the accuracies of genomic predictions ranged from 0.195±0.084 (GBLUP for LMP) to 0.424±0.147 (PBayesB for CW). The average accuracies across traits were 0.327±0.085 (GBLUP), 0.335±0.063 (PBayesA), 0.347±0.093 (PBayesB) and 0.334±0.077 (PBayesCπ), respectively. Notably, parallel Bayesian models were more accurate than GBLUP across six traits. Our study suggested that genomic selections with multiple chains parallel Bayesian models are feasible for slaughter traits in Chinese Simmental cattle. The estimations of direct genomic breeding values using parallel Bayesian methods can offer important insights into improving prediction accuracy at young ages and may also help to identify superior candidates in breeding programs.

## Introduction

Genomic prediction has been widely used to predict breeding values of candidates with genome-wide SNP markers [1], this technology offers great promise to predict genetic merits of selection candidates for economic traits which are difficult or expensive to measure, for instance, traits which may only be measured by sacrificing potential breeding candidates, like carcass traits [2]. With the advance of genomic prediction, the genomic breeding values can be estimated at young ages, and help to promote the genetic progress of breeding in farm animals [3–6].

Carcass traits are important traits in beef cattle, many studies have been conducted to estimate the genomic breeding values of these traits including hot carcass weight, longissimus muscle area, carcass average backfat thickness, lean meat yield and carcass marbling score using BovineSNP50K Beadchip [2, 7–9].

Genomic prediction in beef cattle have been mainly carried out using lower-density SNP chip including Illumina BovineSNP50K Beadchip [2, 8–15], 15K SNP chip and 25K SNP chip[3]. In recent year, several studies have been conducted for genomic prediction using high-density SNP panels [4, 7, 16, 17], and they found genotyping with high density SNP chip can improve the accuracy of genomic prediction for Bayesian methods [18–20]. To obtain higher accuracies from low-density SNP panels, previous studies have attempted to impute lower-density SNPs into high density SNPs data [20–22], and these results suggested that predictive accuracies using imputation data outperformed those using low-density SNPs, while performance (both GBLUP and Bayesian methods) were also influenced by their imputation errors[18].

Many methods have been proposed for genomic prediction including Genomic Best Linear Unbiased Prediction (GBLUP) [23] and Bayesian methods [1, 24]. GBLUP is widely used for its merits of high estimation accuracies and short running time. Bayesian methods, implemented with Markov Chain Monte Carlo (MCMC), show high performances of predictive ability (easy implementation and robustness) in animals and plants breeding [25–27]. However, the iteration process in MCMC requires long computation time. Parallel computing using multiple processing units can shorten the running time of an intensive computational task [28]. Recently, Wu et al. used parallel MCMC to explore high-performance Bayesian computation in animal breeding, and their result suggested parallel MCMC could revolutionize computational tools for breeding programs for animals [15]. In this study, we further extended the parallel computing in genomic prediction by combining multiple chains parallel MCMC with Bayesian models.The objectives of this study were to 1) estimate prediction abilities of genomic selection for slaughter traits in Chinese Simmental beef cattle with GBLUP, parallel Bayesian methods. 2) evaluate the predictive accuracies of these methods. 3) provide valuable insights for application of genomic selection for slaughter traits in Chinese Simmental cattle.

## Methods

### Ethics statement

Animal experiments were approved by the Science Research Department of the Institute of Animal Science, Chinese Academy of Agricultural Sciences (CAAS) (Beijing, China).

### Simulation

We evaluated predictive accuracies and running time of our algorithm in simulation. Here, GPOPSIM software was used to generate simulation dataset including markers and QTLs [29]. Heritability was set to 0.5, the population included 1000 individuals, each chromosome included 10000 markers and the numbers of chromosomes per animal were set to 10. Mutation rate of marker and mutation rate of QTL were both set to 1.25×10^−3^ per locus per generation.

### Animals, phenotypes and SNP data

Analysis data were retrieved from the Dryad Digital Repository: http://datadryad.org/resource/ doi:10.5061/dryad.4qc06 which have been previously described in [30]. Average daily gain weight (ADG) was obtained with body weight gain divided by number of fatten day, the weight gain was the difference between the weight before slaughter and the weight entering in cattle farm. Live weights (LW) were measured before slaughter, and carcass weights (CW) were measured before carcasses being moved to chilling room. Then, carcasses were placed in chilling units for 48 hours before cuts. Retail meat weight (RMW) was estimated as RMW = carcass weight—bone weight—weight of fat covering the carcass. Dressing percentage (DP) were estimated as DP = carcass weight / live weight, and lean meat percentage (LMP) was LMP = (carcass weight- bone weight) / live weight. Summary statistics of these traits including number of animal, mean, standard deviation (SD), minimum and maximum of six traits were listed in Table 1.

**Table 1:** Summary statistics of slaughter traits (number of animal, mean, and standard deviation (SD), maximum and minimum of each trait).

Trait	Num.	Mean±SD	Maximum	Minimum
ADG	1294	0.97 ±0.22	2.41	0.38
LW	1302	505.26±70.73	776	318
CW	1302	271.35 ±45.63	486	162.6
DP	1301	53.56±2.91	68.98	41.03
LMP	1301	45.47 ±3.08	61.56	32.51
RMW	1299	169.94±29.80	280.87	84

ADG: Average daily gain weight (kg), LW: Live weight (kg), CW: Carcass weight (kg), DP: Dressing Percentage (%), LMP: Lean meat percentage (%), RMW: Retail meat weight (kg). Num: Number of animal, Mean±SD: phenotypic mean and standard deviation.

To eliminate potential impact of environmental effects including farm, year of measurement and age for slaughter traits, we corrected phenotypes using the following equation as in [31],

$$y_{ijkm}=u+{Farm}_{i}+{Month}_{j}+{Year}_{k}+e_{ijkm}$$

where, y_ijkm_ is the vector of phenotype, u is the population mean, Farm_i_ is the category of the farm where the animal was raised, Month_j_ is the number of months after birth, Year_k_ is the year of slaughter, e_ijkm_ is the random residual. We processed SNP quality control using PLINK v1.07 [32] software and selected SNPs based on minor allele frequency (>0.05), proportion of missing genotypes (<0.05), Hardy-Weinberg equilibrium (p>10^−6^). 1217 individuals remained after quality control (Table 2) and 671220 SNPs were included in autosomes.

**Table 2:** Birth year distribution for genotyped animals.

Birth year	Animals
2008	91
2009	193
2010	328
2011	276
2012	208
2013	121
Total	1217

### Statistical model

In this study, linear mixture model was used as following,

$$y_{i}=\mu +\sum_{j=1}^{M}Z_{ij}\alpha_{j}+e_{i}$$

where *y*_*i*_ is phenotype for individual *i*, *M* is the number of SNPs, *μ* is the overall mean, *a*_*j*_ is the effect of locus *j*, *Z*_*ij*_ is the SNP genotype code for individual *i* at locus *j* (coded as 0, 1, 2),*e*_*i*_ is the random residual effect for individual *i*.

### BayesA

All loci are assumed to have effects for the trait of interest in BayesA. The prior distribution of effects *α*_*j*_ is assumed to be a normal distribution with a mean 0 and a variance $\sigma_{\alpha_{j}}^{2}$, whereas the prior distribution of $\sigma_{\alpha_{j}}^{2}$ belongs to scaled inverted chi-square distribution, *χ*^-2^(*ν*,S), where S is a scale parameter and *ν* is the number of degrees of freedom. *ν* = 4.012 and S = 0.0020 are used as the prior distribution of $\sigma_{\alpha_{j}}^{2}$. Gibbs sampling is used for the estimation of marker effects and variances [1].

### BayesB

BayesB assumes that some SNPs have zero effect, while other SNPs are assumed to have large effects. Therefore, parameter π is used in BayesB to control whether the locus has a nonzero effect or not.

$$\{\begin{array}{ccc} \sigma_{a_{i}}^{2}=0 & probability & \pi \\ \sigma_{a_{i}}^{2}∼\chi^{-2}(\nu ,S) & probability & \left(1-\pi \right) \end{array}$$

Where *ν* = 4.234 and S = 0.0429 are suggested to yield the mean and variance of $\sigma_{a_{i}}^{2}$. Metropolis Hasting algorithm is used to implement the sampling of variances [1]. In our study, we set π to 0.99.

### BayesCπ

BayesCπ modifies BayesB method by replacing the locus-specific variance components with a common effect variance, and this method assumes an unknown fraction *π* [with uniform (0, 1) prior] of SNP with a nonzero effect, the common variance has a scaled inverse chi-square prior with parameter *ν* = 4.2 and scale factor S, where S is derived as for BayesB [24]. The probability π is treated as an unknown with uniform (0,1) prior, and the effect of a SNP fitted with probability (1-π) comes from a mixture of multivariate student’s t-distributions.

### GBLUP

GBLUP uses mixed model equations with a genomic relationship matrix, assuming a prior normal distribution for SNP markers. The relationship matrix (**A)** based on pedigree is substituted by the genomic relationship matrix (**G)** in GBLUP as defined by VanRaden [23], the G matrix is formulated as follows,

$$G=\frac{ZZ'}{2\sum_{i=1}^{n}q_{i}(1-q_{i})},$$

where *n* is the number of loci, *q*_*i*_ is the frequency of an allele of the marker *i*, and **Z** is a centered incidence matrix of SNP effects, corrected for allele frequencies [23].

### Implementation of multiple chains parallel Bayesian prediction

MCMC includes two steps, sampling in burn-in and sampling after burn-in. In multiple chains parallel MCMC, sampling in burn-in should be implemented sequentially and parallelization can only happen in sampling after burn-in [33]. Thus, sampling in burn-in in parallel MCMC requires the same number of iteration as that in sequential MCMC. In experiments, the number of chains used in parallel Bayesian models in simulation were set to 1 chain (sequential models) and 9 different multiple (2, 4, 6, 8, 10, 12, 14, 16, 18) chains, while the number of chains was set to 16 on real dataset. The maximal iteration of MCMC (both on simulation dataset and real dataset) was set to 50000 with 5000 burn-in.

**Fig 1:**
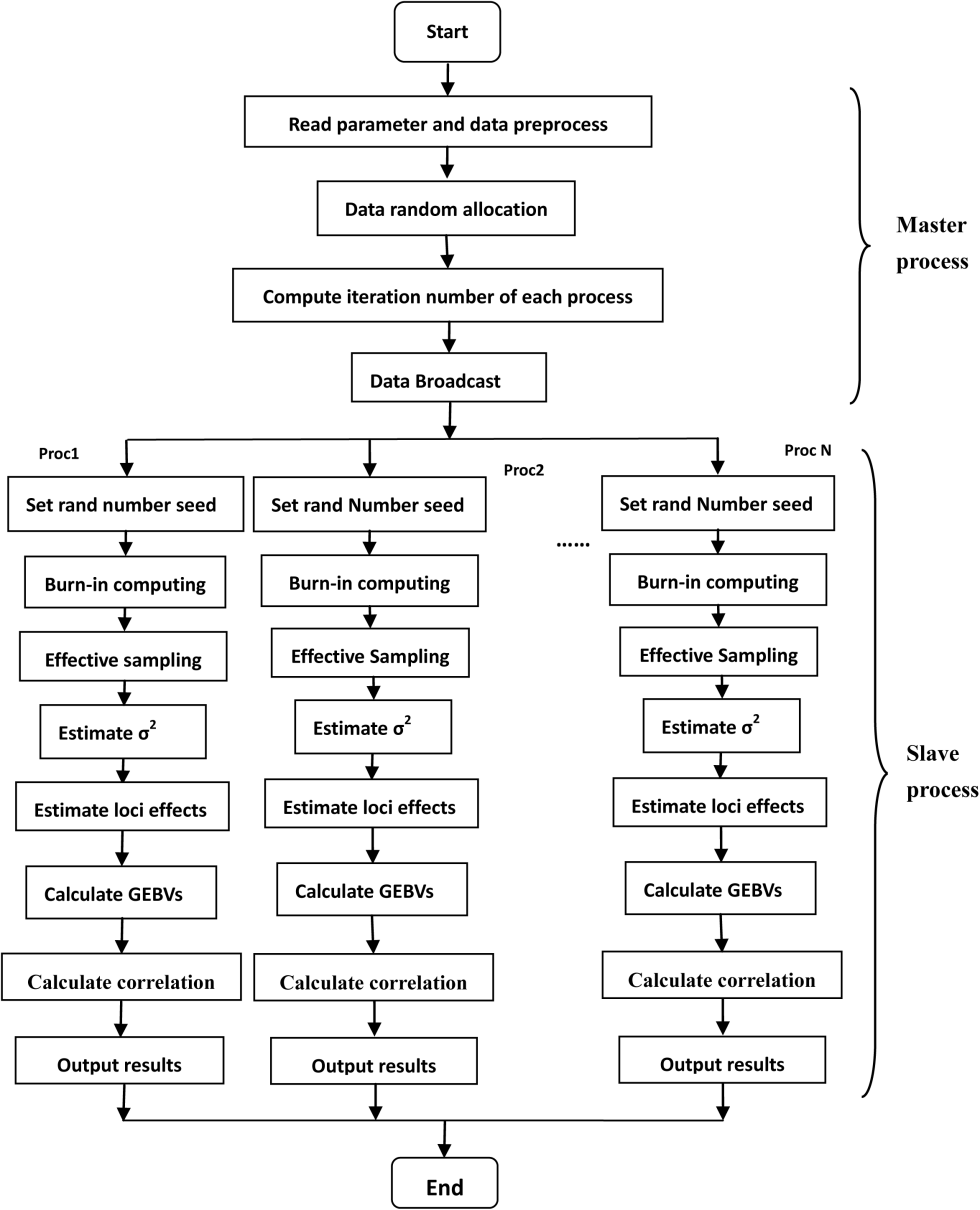
Workflow of multiple chains parallel Bayesian genomic prediction. Proc1: Process 1, Proc2: Process 2, ProcN: Process N. σ^2^: variance of normal distribution for estimated effects.

In parallel computing, computing tasks are executed in process, and each process is dispatched to one computing core. In our study, computation in Bayesian model was divided into sequential part and parallel parts, sequential part was implemented by master process, and its tasks included loading data from files, initializing parameters, broadcasting data and parameters to parallel parts. Parallel parts were implemented by slave processes independently, slave processes computing tasks included random number seed setting, burn-in computing, estimating locus effect and calculating GEBVs (Fig 1).

### Multiple chains convergence diagnosis

Multiple chains convergence diagnosis followed Gelman and Rubin’s method [34]. $\hat{R}$ is the shrink factor, if $\sqrt{\hat{R}}>1$, the chains don’t converge, if $\sqrt{\hat{R}}\approx 1$, the chains converge.

### GEBV calculation

GEBV is calculated as the sum of all SNP effects according to marker genotypes and genotype effects. Just as the following equation.

$$GEBV_{i}=\sum_{j}Z_{ij}g_{j}$$

where *GEBV*_*i*_ is the genomic estimated breeding value of animal *i*, *Z*_*ij*_ is a genotype for SNP *j* of animal *i*, and *g*_*j*_ is the estimated effect of the *j*th SNP locus.

### Cross-validation procedure

To evaluate the predictive accuracies, random masking cross-validation method was used in this study [13]. A total of 1217 Simmental cattle were divided into validation set and training set. Phenotypes of animals in the validation set were assumed unknown. Five-fold cross validation was used to assess the accuracies of prediction, and 1217 individuals were randomly partitioned into five groups. In each time, about one-fifth of 1217 Simmental were randomly picked out as the validation set and the remaining individuals were used as the training set. For each trait, the procedure was repeated 10 times and the average value was calculated as the GEBV.

### Predictive criterion

To remove the influence of the heritability for predictive ability, we used Pearson's correlation between GEBVs and corrected phenotypes divided by square root of heritability ($r_{\hat{g},\hat{y}}/\sqrt{h^{2}}$), here, $r_{\hat{g},\hat{y}}$ was the correlation between GEBVs and corrected phenotypes, $\hat{y}$ was the vector of corrected phenotype in validation set and $\hat{g}$ was the vector of GEBVcalculated with SNP data in validation set and effects obtained in training set [8]. Moreover, we compared results using average values and standard deviations of predictive accuracies.

### Computer system

Our experiments were conducted on HP ProLiant DL585 G7 server, which was equipped with AMD Opteron 6344(2.6GHz) CPU, 272G Memory and L2 cache size 4M, L3 cache size 16M. We wrote programs in C language within Message Passing Interface (MPI) system, MPICH2 is an open source MPI implementation and a standard for message-passing in parallel computing, it is available freely (http://www.mpich.org/downloads). The Integrated Development Environment we used is Dev-C++ 5.1, which is published freely (http://www.bloodshed.net/index.html).

## Result

### Results using simulation dataset

**Fig 2:**
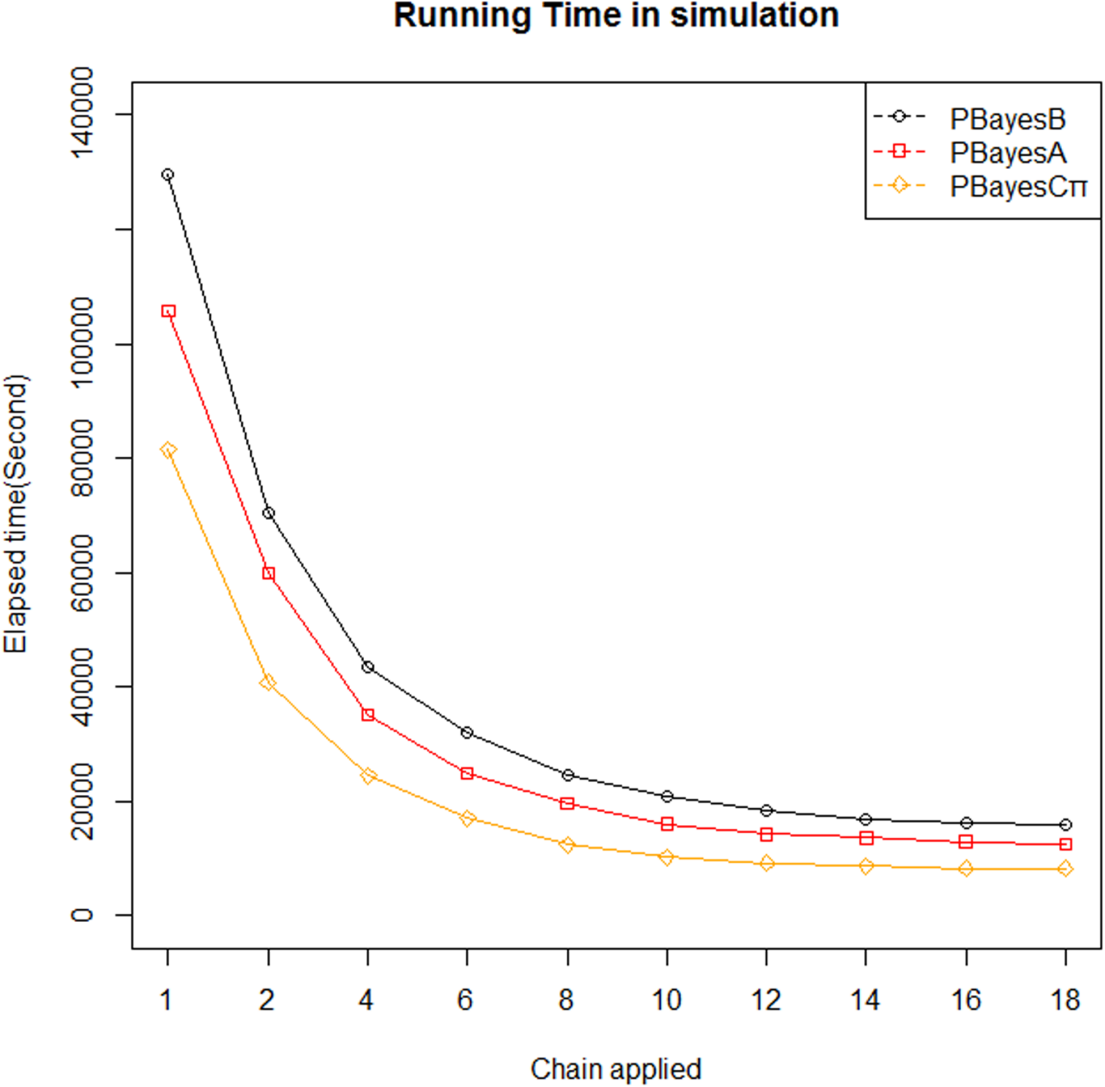
Comparisons of running time in simulation. Axis x indicates chains number used in parallelization and axis y indicates running time.

Predictive accuracies using multiple chains were shown in Table 3. For PBayesA, PBayesB or PBayesCπ, there were tiny differences of predictive accuracies among different chains’ results from the same parallel Bayesian method, the maximal difference was from PBayesCπ, where the largest accuracy (0.868763 using 10 chains) was 0.18% (percent point difference) higher than the smallest accuracy (0.86716 using 4 chains), and the minimal difference was from PBayesA,where the largest accuracy (0.836904 using 6 chains) was 0.04% higher than the smallest accuracy (0.836559 using 8 chains). In this study, the descending order of predictive accuracies for four methods were found (PBayesB> PBayesCπ>PBayesA>GBLUP) in simulation. We evaluated the running time across PBayesA, PBayesB and PBayesCπ in simulation, and we found the running time reduced obviously for the three parallel Bayesian methods with increase of chain number (Fig 2).

**Table 3:** Predictive accuracies using four methods using PBayesA, PBayesB, PBayesCπ and GBLUP in simulation.

	PBayesA	PBayesB	PBayesCπ	GBLUP
1 ch	0.836777	0.887837	0.867312	0.829173
2ch	0.836731	0.887741	0.867161	
4ch	0.836797	0.887449	0.867160	
6ch	0.836904	0.888563	0.868017	
8ch	0.836559	0.888305	0.868319	
10ch	0.836770	0.888232	0.868763	
12ch	0.836862	0.888997	0.868347	
14ch	0.836814	0.888535	0.868426	
16ch	0.836844	0.888852	0.868385	
18ch	0.836820	0.888823	0.868210	

ch means chains used in experiments, GBLUP: Genomic Best Linear Unbiased Prediction, PBayesA: multiple chains parallel BayesA, PBayesB: multiple chains parallel BayesB, PBayesCπ: multiple chains parallel BayesCπ.

### Predictive accuracies

**Fig 3:**
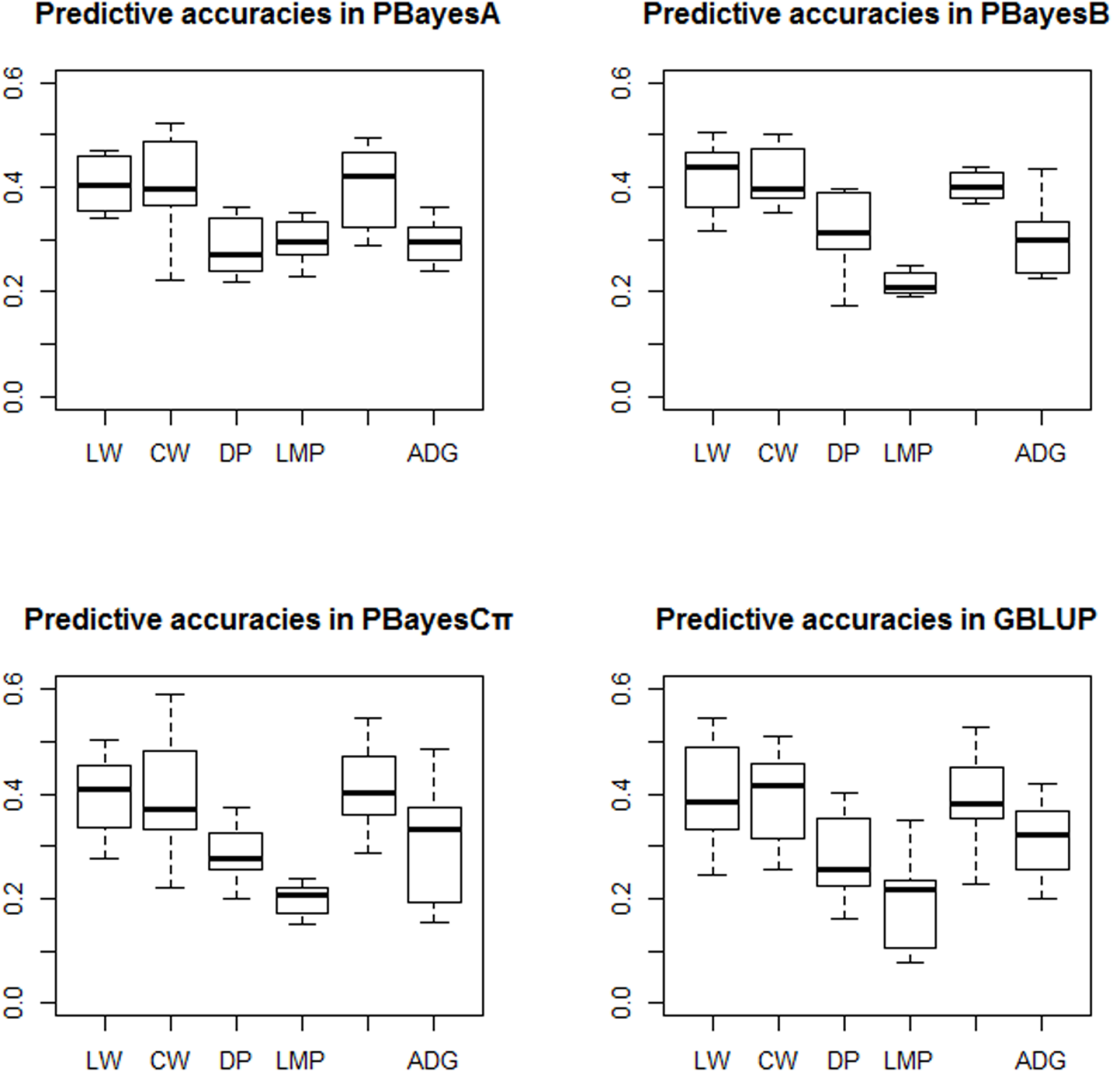
Predictive accuracies using GBLUP, PBayesA, PBayesB and PBayesCπ for slaughter traits in Chinese Simmental cattle. LW: Live weight (kg), CW: Carcass weight (kg), DP: Dressing Percentage (%), LMP: Lean meat percentage (%), RMW: Retail meat weight (kg), ADG: Average daily gain weight (kg). PBayesA: multiple chains parallel BayesA, PBayesB: multiple chains parallel BayesB, PBayesCπ: multiple chains parallelBayesCπ. GBLUP: Genomic Best Linear Unbiased Prediction.

In this study, we calculated heritabilities of slaughter traits (Table 4) using restricted maximum likelihood (REML) based on animal model. Random masking cross-validation method was applied to assess the predictive accuracies of slaughter traits in Simmental cattle population. In general, the predictive accuracies for most traits were slightly different between parallel Bayesian models and GBLUP. Accuracies of genomic predictions were ranged from 0.195±0.084 (GBLUP for LMP) to 0.424±0.147 (PBayesB for CW). The average accuracies across traits were 0.327±0.085 for GBLUP, 0.335±0.063 for PBayesA, 0.347±0.093 for PBayesB and 0.334±0.077 for PBayesCπ (Table 4). Prediction accuracies among the four methods for six traits were presented in Fig 3.

**Table 4:** Heritabilities estimation and predictive accuracies of GEBVs for slaughter traits in Chinese Simmental cattle.

Trait	h^2^	Average value (standard deviation) of correlations				Average value (standard deviation) of correlations divided by square root of heritability			
		GBLUP	PBayesA	PBayesB	PBayesCπ	GBLUP	PBayesA	PBayesB	PBayesCπ
LW	0.37	0.236 (0.060)	0.247 (0.032)	0.257 (0.040)	0.242 (0.044)	0.388 (0.099)	0.405 (0.053)	0.423 (0.066)	0.398 (0.072)
CW	0.45	0.266 (0.059)	0.271 (0.057)	0.285 (0.098)	0.268 (0.080)	0.397 (0.089)	0.404 (0.085)	0.424 (0.147)	0.399 (0.119)
DP	0.16	0.111 (0.031)	0.114 (0.021)	0.124(0.045)	0.114 (0.021)	0.277 (0.076)	0.285 (0.053)	0.311 (0.113)	0.285 (0.052)
LMP	0.14	0.073 (0.031)	0.081 (0.025)	0.080 (0.009)	0.074 (0.010)	0.195 (0.084)	0.216 (0.066)	0.214 (0.023)	0.198 (0.028)
RMW	0.43	0.258 (0.058)	0.265 (0.050)	0.265 (0.021)	0.271 (0.050)	0.393 (0.088)	0.404(0.076)	0.404 (0.032)	0.413 (0.076)
ADG	0.47	0.214 (0.052)	0.203 (0.029)	0.210 (0.120)	0.213 (0.079)	0.312 (0.076)	0.297 (0.042)	0.306 (0.175)	0.311 (0.115)
mean		0.193 (0.049)	0.197 (0.036)	0.204 (0.056)	0.197 (0.047)	0.327 (0.085)	0.335 (0.063)	0.347 (0.093)	0.334 (0.077)

LW: Live weight (kg), CW: Carcass weight (kg), DP: Dressing Percentage (%), LMP: Lean meat percentage (%), RMW: Retail meat weight (kg), ADG: Average daily gain weight (kg).

For most traits, parallel Bayesian methods resulted in slightly higher accuracies than GBLUP. For LW, CW and DP, PBayesB performed best among these four methods, and the percentage point differences between PBayesB and GBLUP were 9.02% for LW, 6.80% for CW and 12.27% for DP respectively. For LMP, PBayesA showed higher predictive accuracy than GBLUP (10.77%). For RMW, we found PBayesCπ, PBayesB and PBayesA were superior to GBLUP, while GBLUP was superior over parallel Bayesian methods for ADG.

### Posterior samples of residual variance

**Fig 4:**
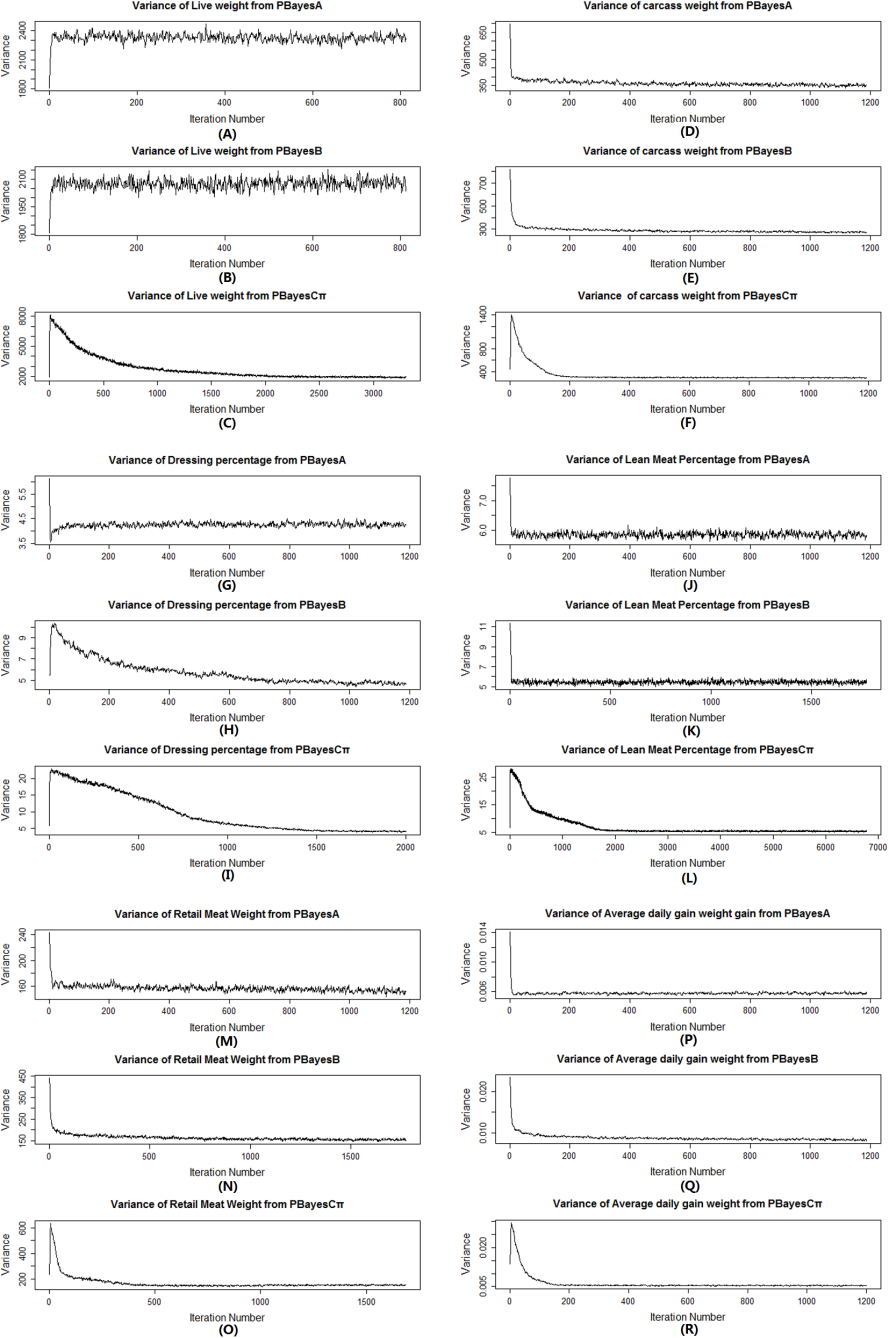
Trace plots of posterior samples of residual variancesin burn-in (from start to equilibrium) from multiple chains parallel Bayesian models for 6 traits. (A)Trace plots for live weight, (B) Trace plots for carcass weight, (C) Trace plots for dressing percentage,(D) Trace plots for lean meat percentage (E) Trace plots for retail meat weight, (F) Trace plots for average daily weight gain.PBayesA: multiple chains parallel BayesA, PBayesB: multiple chains parallel BayesB, PBayesCπ: multiple chains parallel BayesCπ.

Posterior samples of residual variance were used in convergence diagnosis analysis as described in previous study [15]. The largest percent point difference among PBayesA, PBayesB and PBayesCπ was found for ADG, the difference happened between PBayesB and PBayesCπ (30.91%), posterior samples of residual variance approached 0.0061 (PBayesA), 0.0072 (PBayesB) and 0.0055 (PBayesCπ) which were shown in Fig 4P–Fig 4R.While the slightest percent point difference was found in RMW, the difference happened between PBayesA and PBayesCπ (1.33%), posterior samples of residual variance were 152 (PBayesA),154 (PBayesB) and 150 (PBayesCπ) (Fig 4M–Fig 4O). For LW, CW, DP and LMP,we also observed slight differences for posterior samples of the residual variances using PBayesA, PBayesB and PBayesCπ (Fig 4A–4L).

### Convergence diagnose of multiple chains

**Fig 5:**
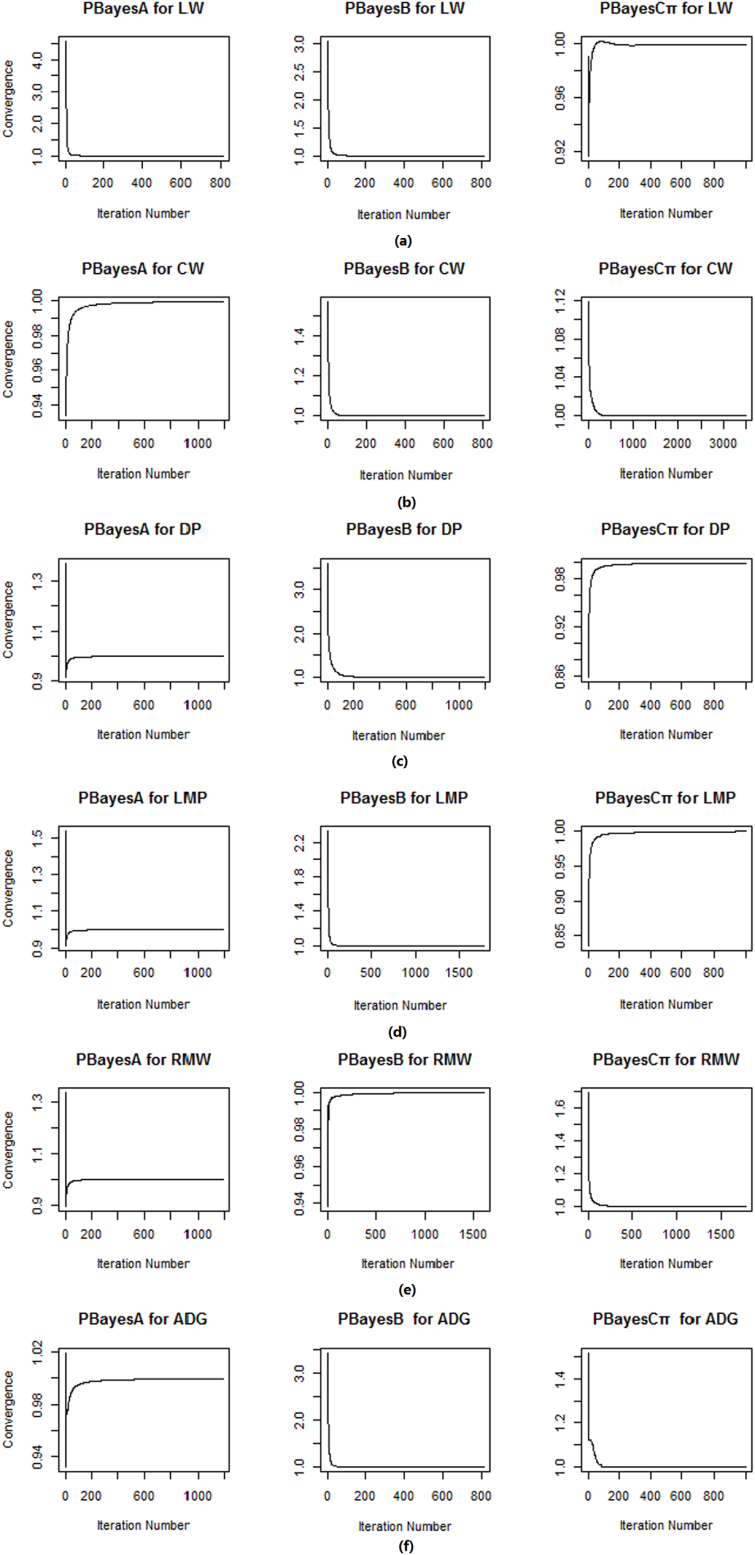
Trace plot of convergence of multiple chains (from start to equilibrium) for 6 traits. (a) Parallel Bayesian models for LW, (b) Parallel Bayesian models for CW, (c) Parallel Bayesian models for DP, (d) Parallel Bayesian models for LMP, (e) Parallel Bayesian models for RMW, (f) Parallel Bayesian models for ADG.PBayesA: multiple chains parallel BayesA, PBayesB: multiple chains parallel BayesB, PBayesCπ:multiple chains parallel BayesCπ.

In multiple Markov chains parallel Bayesian genomic prediction, convergence diagnose helps determine the equilibrium of MCMCs. With convergence diagnosis criterion proposed by Gelman and Rubin [34], we assessed the convergence of multiple chains for the genomic prediction of slaughter traits, and we observed the shrink factors of PBayesA, PBayesB and PBayesCπ quickly approached 1.00 for six traits (Fig 5), which indicated multiple chains converged in parallel Bayesian models.

## Discussion

In this study, we carried out genomic prediction for slaughter traits using GBLUP and Bayesian models in Chinese Simmental cattle. In the last decade, beef cattle have been selected for various economic traits such as growth [2, 7–9], carcass [10–14], meat [35] and reproduction [35, 36]. To maximize the economic benefits of beef cattle reproduction, selection for economically important traits is desirable. Therefore, slaughter traits (live weight, carcass weight, dressing percentage, lean meat percentage, retail meat weight andaverage daily gainweight) have been mostly focused by beef cattle industry.

Genomic predictions have aroused scientists’ interests for merits of robustness, easy implementation and higher predictive capability. The intensive computing of Bayesian models may require days, weeks, or even months of computing time on personal computers or workstations [15] and this computational burden is the most obvious obstacle for its application in animals and plants breeding. Stranden et al. used parallel preconditioned conjugate gradient method to estimate breeding values in Finnish dairy cattle, running time using four processors was obviously reduced in contrast to that of sequential mode [37]. Using theoretical and experiment analyses, Wu et al. found obvious reduction of running time for experimental results using parallel MCMC method in breeding estimation [15]. Running time reduction of PBayesA, PBayesB and PBayesCπ using simulation dataset (Fig 2) were consistent with previous studies [15, 37]. In current study, we used parallel BayesA, BayesB and BayesCπ to estimate genomic breeding values for slaughter traits by dividing the heavy computing task into several segments, and our results provided valuable insights for application of genomic selection using parallel MCMC for these traits in Chinese Simmental cattle.

### Model comparisons

GBLUP shows obvious superiority over Bayesian models on computing time, for instance, the time taken in GBLUP is less than one minute for each of the 5-fold cross validation, while 3 days were required in the genomic prediction using Bayesian models. The reason for obvious difference in computing time may be caused by model, population size and marker number. In GBLUP, genomic matrix calculation is a time consuming process, and for a population with certain number of individuals and genotyped data, genomic matrix calculation is implemented only once and the result can be reused in genomic prediction for other traits in the same population. While in Bayesian models, effect of each locus was estimated with MCMC method, the MCMC sampling procedure should be implemented thousands of times.

Bayesian methods can appropriately model the architecture of QTL effects within the genome, especially for traits that possess large effect QTLs [13]. It has previously been observed that the genomic predictive ability depends on attributes of genetic architecture of the trait, population size and particular model. We observed the predictive accuracies of Bayesian models were slightly different for 6 traits using 3 parallel Bayesian methods, and the performance of accuracy was PBayesB > PBayesA > PBayesCπ.

### Genomic prediction methods

In this study,we found parallel Bayesian models outperformed GBLUP for most traits. Previous studies have suggested that GBLUP outperformed Baysian methods using low-density chip including 15K SNP chip [3] and 25K SNP chip [3, 38]. In contrast, Erbe et al.suggested Bayesian method (Bayes R) was superior over GBLUP after analyzing genomic selection in dairy cattle using imputed high-density panel, and their finding also implied Bayesian methods may take full advantage of the increased marker density [25]. Bayesian methods outperformed GBLUP for traits controlled by several SNPs with large effects, while GBLUP performed better for those traits which were not controlled by large effects SNPs. This could be explained that the genetic architecture of ADG was different from other traits. Our results also suggested that GBLUP was suitable for ADG, while PBayesA, PBayesB and PBayesCπ were suitable for other traits in Chinese Simmental cattle population.

### Accuracies of genomic predictions

To comprehensively evaluate the accuracies of estimated breeding values among PBayesA, PBayesB and PBayesCπ, we ran different multiple chains in simulation data set using the three methods. For the same Bayesian methods, we found that slight difference among predictive accuracies of sequential Bayesian method and multiple chains parallel Bayesian methods, this indicated that parallel Bayesian methods can generate equivalent accuracies comparing to that of sequential Bayesian methods. In general, the descending order of predictive accuracies in simulation was PBayesB > PBayesCπ > PBayesA > GBLUP.

Accuracies of genomic prediction can be impacted by the model, heritability of the trait, the size of the reference population, the density of the SNP panel and level of LD [2]. Previous study revealed that traits with a larger number of genotyped animals and higher heritability generated the higher accuracy of GEBV [7]. For six studied traits, we found obvious differences among the estimated heritabilities, heritabilities of LW (h^2^ = 0.37), CW (h^2^ = 0.45), RMW (h^2^ = 0.43) were higher than those of DP (h^2^ = 0.16) and LMP (h^2^ = 0.14), while predictive accuracies for LW, CW and RMW were higher than those for DP and LMP, and our findings were consistent with previous studies [7, 39]. Notably, we found the heritability for ADG was 0.47, and the predictive accuracies were 0.297±0.042 (for PBayesA), 0.306±0.175 (for PBayesB), 0.311±0.115 (for PBayesCπ) and 0.312±0.076 (for GBLUP), thus, our results suggested that density of the SNP panel, level of LD and the model may also have important impacts on predictive accuracies.

Compared to accuracies of CW in previous studies [7, 39], our results (0.397±0.089 for GBLUP, 0.404±0.085 for BayesA, 0.424±0.147 for BayesB and 0.399±0.119 for BayesCπ) was higher than those of Nellore (0.37±0.053 for Bayesian ridge regression, 0.36±0.058 for BayesC and 0.37±0.056 for Bayesian Lasso) [39], Angus (0.16 for GBLUP), Shorthorn (0.19 for GBLUP), Brahman (0.28 for GBLUP) and Santa Gertrudis (0.29 for GBLUP) cattle, Hereford (0.32 for GBLUP), Belmont Red (0.33 for GBLUP), and was similar to that of Murray Grey (0.39 for GBLUP) cattle [7].For ADG, accuracies of our results (0.312±0.076 for GBLUP, 0.297±0.042 for PBayesA, 0.306±0.175 for PBayesB, 0.311±0.115 for PBayesCπ) were higher than Angus (0.24 for GBLUP and for Bayes R), Belmont Red (0.24 for GBLUP and 0.18 for BayesR), Brahman crosses (0.13 for GBLUP and 0.27 for BayesR), Santa Gertrudis (0.21 for GBLUP and 0.23 for BayesR) [7]. This indicated that genomic selection using multiple chains parallel Bayesian models was suitable for genomic prediction for LW, CW, RMW, DP and LMP in Chinese Simmental beef cattle.

### Multiple chains convergence diagnosis

In multiple chains MCMC, effective sampling should happen when chains converges. During the evaluation of convergence of multiple chains, we observed sampling results from start to the point when chains being in equilibrium in burn-in step. Sampling results and shrink factors in equilibrium were stable and we omited part of trace plots in equilibrium across traits.

For multiple chains MCMC, each one started with different initial value, and all chains should converge after a certain number of iteration. We used Gelman and Rubin’s method [34] to evaluate multiple chains’ convergence state. The convergence was examined using posterior samples of the residual variance collected from each chain. Posterior samples showed slight differences among parallel Bayesian models for the same traits and trace plots of posterior samples of the residual variance indicated that most chains tended to stabilize after 2000 iterations (Fig 4).

We assessed parallel Bayesian models for six traits, and all shrink factors approached 1 (Fig 5(A)–5(F)). Wu et al. suggested that a burn-in of 3000 iterations being more appropriate [15], our results showed that shrink factors approached 1 with less than 3000 iteration, this finding suggested that multiple chains MCMC converged obviously in Simmental beef cattle dataset.

## Conclusions

Our study demonstrated that it is feasible for the application of parallel genomic prediction for slaughter traits in Chinese Simmental beef cattle. Our results indicated that parallel BayesB outperformed GBLUP, parallel BayesA and parallel BayesCπ. Moreover, the predictive accuracies of parallel Bayesian models were more accurate than GBLUP for most traits and these methods are interest for the future application of genomic selection in farm animals.
